# The interplay of insulin resistance and systemic inflammation on cancer history: a nationally representative cross-sectional study from NHANES

**DOI:** 10.1186/s40842-026-00335-y

**Published:** 2026-11-04

**Authors:** Xiaorui Wang

**Affiliations:** Sanya University, 191 Xueyuan Road, Jiyang District, Sanya, Hainan Province P.R. China

**Keywords:** Insulin resistance, Neutrophil-to-lymphocyte ratio, Cancer history, National health and nutrition examination survey, Effect modification

## Abstract

**Aim:**

To investigate whether systemic inflammation, measured by the neutrophil-to-lymphocyte ratio (NLR), modifies the cross‑sectional association between insulin resistance (IR, assessed by HOMA-IR) and overall cancer history in a nationally representative population.

**Methods:**

We conducted a cross-sectional analysis utilizing data from the 2017–2018 National Health and Nutrition Examination Survey (NHANES), which employs a complex, multistage probability sampling design. All analyses accounted for the complex survey design and sampling weights to ensure national representativeness. Weighted multivariable logistic regression was used to assess the cross‑sectional association, with an interaction term between HOMA-IR and NLR.

**Results:**

Among 3,746 adults, after adjusting for age, sex, and BMI, HOMA-IR was not independently associated with cancer history (OR = 1.00, 95% CI 0.98–1.02). The HOMA-IR × NLR interaction term was not statistically significant (P for interaction = 0.359), indicating no significant effect modification by NLR.

**Conclusion:**

In this nationally representative sample, we found no evidence that systemic inflammation, as measured by NLR, amplifies the cross‑sectional association between IR and overall cancer history. The potential synergistic effect may be confined to specific high-risk subgroups or inflammatory pathways.

**Supplementary Information:**

The online version contains supplementary material available at https://doi.org/10.1186/s40842-026-00335-y.

## Introduction

The global epidemics of obesity, type 2 diabetes mellitus (T2DM), and cardiovascular diseases (CVD) share a common pathophysiological soil characterized by insulin resistance (IR) and chronic low-grade inflammation [1]. These intertwined metabolic and inflammatory disturbances are not only pivotal in the development and progression of T2DM and atherosclerosis but have also been implicated as significant drivers of increased cancer history among affected individuals [2]. This convergence of pathologies underscores a critical public health challenge: managing the systemic complications that extend beyond the primary cardiometabolic disorders.

IR, a hallmark of T2DM and metabolic syndrome, fuels a state of hyperinsulinemia that can directly promote tumorigenesis through insulin and insulin-like growth factor-1 (IGF-1) signaling pathways [3]. Concurrently, IR perpetuates a state of systemic inflammation, creating a vicious cycle that further aggravates metabolic dysfunction and tissue damage [1]. The neutrophil-to-lymphocyte ratio (NLR) has emerged as a robust, stable hematological marker of this systemic inflammatory burden and has been independently associated with both future cardiovascular events [4] and overall mortality [5]. This positions NLR at the intersection of metabolic dysregulation and its downstream sequelae.While the independent roles of IR and inflammation in cardiometabolic and oncological diseases are well-recognized, epidemiological evidence directly testing their interactive effect on disease risk in population settings remains scarce. Specifically, it is unknown whether the systemic inflammatory state, a modifiable risk factor, amplifies the carcinogenic risk associated with IR in the general population—a population that includes the vast reservoir of individuals with undiagnosed prediabetes or subclinical metabolic syndrome.

Therefore, utilizing data from the nationally representative National Health and Nutrition Examination Survey (NHANES), this study aims to investigate the interplay between IR (assessed by HOMA-IR) and systemic inflammation (measured by NLR) on the risk of a history of cancer. By testing this interaction, we seek to translate the conceptual framework of metabolic-inflammatory crosstalk to the population level. Our findings aim to provide insights relevant not only for cancer prevention but also for the comprehensive risk stratification of individuals along the spectrum of insulin resistance—a core feature of diabetes and cardiovascular disease.

## Methods

### Study design and population

**Fig. 1:**
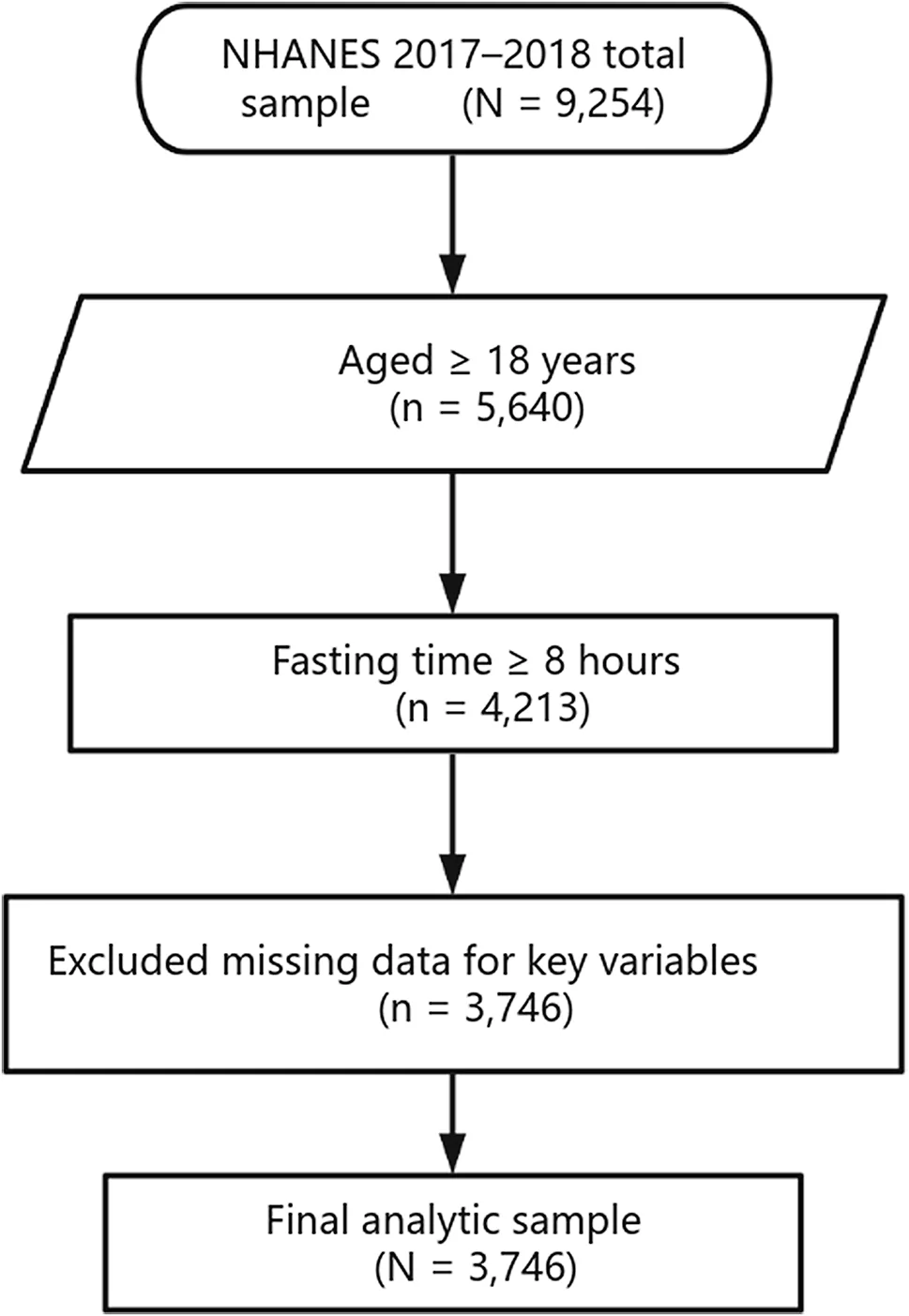
Participant flow diagram. NHANES, national health and nutrition examination survey. the final analytic sample comprised 3,746 adults after applying the following exclusion criteria: age < 18 years, fasting time < 8 h, and missing data for key variables (HOMA-IR, NLR, or cancer history)

This cross-sectional analysis used data from the 2017–2018 National Health and Nutrition Examination Survey (NHANES). We included adults with a reported fasting time ≥ 8 h. After exclusions for missing data, the final analytic sample comprised 3,746 individuals. A detailed participant flow diagram is provided in Fig. 1.

### Variable definitions

Primary Exposure (Insulin Resistance): Quantified bythe Homeostasis Model Assessment index (HOMA-IR): [Fasting insulin (µU/mL) × Fasting glucose (mg/dL)] / 405.

Effect Modifier (Systemic Inflammation): Theneutrophil-to-lymphocyte ratio (NLR)wascalculated as the absolute neutrophil count divided by the absolute lymphocyte count.2.

Study Outcome (Cancer History): Definedas a self-reported, physician-diagnosed history of any cancer.

Covariates: Age, sex, race/ethnicity, body mass index, smokingstatus, and alcohol use.

### Statistical analysis

All analyses were performed in R (version 4.5.2) using the survey package, incorporating sampling weights, strata, and clusters to obtain nationally representative estimates as per NHANES guidelines 6.

Weighted Descriptive Statistics: Means (SE) for continuous variables and proportions (SE) for categorical variables were calculated.

Main Effect Model: Weighted multivariable logistic regression assessed the cross‑sectional association between HOMA-IR (continuous) and cancer history, adjusted for covariates.

Interaction Analysis: An interaction term (HOMA-IR × NLR) was added to test for effect modification.

Missing data for covariates were handled by complete‑case analysis, as the proportion of missing values was small (< 5%). No imputation was performed.

All tests were two-sided (α = 0.05).

### Ethical considerations

The NHANES study protocol was approved by the National Center for Health Statistics Research Ethics Review Board. Informed consent was obtained from all participants.

## Results

### Characteristics of the study population

This study ultimately included 3,746 adults with a fasting time of ≥ 8 h. Table 1 presents the nationally representative characteristics of this population after applying complex sampling weights. The mean age of the study population was 44.2 years (Standard Error, SE: 0.58). The weighted mean of the insulin resistance marker HOMA-IR was 3.80 (SE: 0.16), and the weighted mean of the inflammation marker NLR was 2.07 (SE: 0.04). The total prevalence of physician-diagnosed cancer was 8.1% (SE: 0.6%).Differences in baseline characteristics between participants with and without a history of cancer were compared using weighted statistics, with results presented in Table 1.

**Table 1:** Weighted baseline characteristics of the study population (*N* = 3,746)

Characteristic	Overall *N* = 238,945,937^1^	0 *N* = 213,279,709^1^	1 *N* = 25,666,228^1^	*p*-value^2^
RIDAGEYR	48.24 (17.22)	46.35 (16.60)	63.95 (13.99)	< 0.001
RIAGENDR				0.024
1	115,304,246 (48%)	105,576,232 (50%)	9,728,013 (38%)	
2	123,641,691 (52%)	107,703,477 (50%)	15,938,215 (62%)	
RIDRETH1				< 0.001
1	23,033,492 (9.6%)	21,769,788 (10%)	1,263,704 (4.9%)	
2	16,013,118 (6.7%)	15,052,318 (7.1%)	960,800 (3.7%)	
3	147,804,092 (62%)	127,147,368 (60%)	20,656,724 (80%)	
4	27,029,536 (11%)	25,278,753 (12%)	1,750,783 (6.8%)	
5	25,065,700 (10%)	24,031,482 (11%)	1,034,217 (4.0%)	
Unknown	203,961,121	182,809,195	21,151,926	
MCQ035				0.5
1	21,107,401 (60%)	18,372,292 (60%)	2,735,109 (61%)	
2	12,262,747 (35%)	10,514,333 (35%)	1,748,414 (39%)	
9	1,614,668 (4.6%)	1,583,889 (5.2%)	30,779 (0.7%)	
Unknown	203,961,121	182,809,195	21,151,926	
MCQ040				< 0.001
1	9,845,235 (47%)	9,306,160 (51%)	539,075 (20%)	
2	11,238,492 (53%)	9,042,458 (49%)	2,196,034 (80%)	
9	23,674 (0.1%)	23,674 (0.1%)	0 (0%)	
Unknown	217,838,536	194,907,417	22,931,119	
BMXBMI	29.60 (7.21)	29.68 (7.34)	28.97 (5.95)	0.8
Unknown	2,649,658	2,295,117	354,542	
HOMA_IR	3.93 (7.37)	3.89 (6.84)	4.25 (10.85)	0.7
Unknown	4,184,816	3,736,735	448,081	
NLR	2.13 (1.15)	2.07 (1.11)	2.62 (1.33)	< 0.001
Unknown	160,652	0	160,652	

^1^Mean (SD); n (%)

^2^Design-based KruskalWallis test; Pearson’s X^2: Rao & Scott adjustment

SE, standard error. Data are presented as weighted mean (SE) for continuous variables and weighted percentage (SE) for categorical variables

### Cross‑sectional association of insulin resistance, inflammation, and cancer

Table 2 presents the results of the weighted multivariable logistic regression model (Model 1), which examined the independent cross‑sectional association of HOMA-IR with cancer history after adjusting for age, sex, and BMI.

In the adjusted model, a one-unit increase in HOMA-IR was not associated with cancer history (Odds Ratio, OR = 1.00; 95% Confidence Interval, CI: 0.98–1.02; *P* = 0.941). Age was the strongest predictor of cancer history, with a 1-year increase in age associated with an approximately 7% higher risk (OR = 1.07; 95% CI: 1.06–1.09; *P* < 0.001). Males had a significantly higher cancer history compared to females (OR = 1.72; 95% CI: 1.10–2.68; *P* = 0.020). Body Mass Index (BMI) showed no significant cross‑sectional association with cancer history (*P* = 0.399).

**Table 2:** Model 1: cross‑sectional association Analysis of Insulin Resistance (HOMA-IR) with cancer history

Variable	Beta Coefficient (SE)	Odds Ratio (OR)	95% Confidence Interval	*P*-value
Insulin Resistance(HOMA-IR)	0.000667(0.00885)	1.00	0.98–1.02	0.941
Age (per 1-year increase)	0.0692 (0.00684)	1.07	1.06–1.09	< 0.001
Sex (Male vs. Female)	0.541 (0.199)	1.72	1.10–2.68	0.020
Body Mass Index (BMI)	-0.0160 (0.0182)	0.98	0.95–1.02	0.399
Intercept	-6.39 (0.638)	-	-	< 0.001

SE, standard error; OR, odds ratio; CI, confidence interval. The model was adjusted for age, sex, and BMI

### Analysis of effect modification by inflammation

To examine whether systemic inflammation (NLR) modifies the above cross‑sectional association, an interaction term between HOMA-IR and NLR was introduced into the model (Model 2). The complete results are shown in Table 3.

In the model including both the main effects of HOMA-IR and NLR and their interaction term, the HOMA-IR × NLR interaction term did not reach statistical significance (interaction coefficient = 0.00808, SE = 0.00835; P for interaction = 0.359). This indicates that in this nationally representative adult sample, no evidence was found to suggest that NLR level significantly modifies the strength of the cross‑sectional association between HOMA-IR and overall cancer history. The direction and significance of cross‑sectional associations for other variables in the model remained largely consistent with Model 1.

**Table 3:** Model 2: analysis of effect modification by inflammation (NLR) on the cross‑sectional association between insulin resistance and cancer

Variable	Beta Coefficient (SE)	Odds Ratio (OR)	95% Confidence Interval	*P*-value
Insulin resistance (HOMA-IR)	-0.0327 (0.0350)	0.97	0.90–1.04	0.374
Inflammation Marker (NLR)	0.102 (0.123)	1.11	0.87–1.41	0.429
HOMA-IR × NLR	0.00808(0.00835)	1.01	0.99–1.03	0.359
Age (per 1-year increase)	0.0669 (0.00664)	1.07	1.06–1.09	< 0.001
Sex (Male vs. Female)	0.516 (0.203)	1.68	1.11–2.53	0.031
Body Mass Index (BMI)	-0.0133 (0.0196)	0.99	0.95–1.03	0.515
Intercept	-6.47 (0.644)	-	-	< 0.001

SE, standard error; OR, odds ratio; CI, confidence interval; NLR, neutrophil-to-lymphocyte ratio. The model included the main effects of HOMA-IR and NLR, their interaction term, and was adjusted for age, sex, and BMI

## Discussion

This study utilized nationally representative NHANES data to test the hypothesis that systemic inflammation, as measured by the neutrophil-to-lymphocyte ratio (NLR), modifies the cross‑sectional association between insulin resistance (quantified by HOMA-IR) and overall cancer history. The following sections summarize the key findings, provide an in-depth interpretation in the context of existing literature, analyze the study’s strengths and limitations, and suggest directions for future research.

### Summary of key findings

The primary finding of this study is clear: no significant effect modification by systemic inflammation (NLR) on the cross‑sectional association between insulin resistance (HOMA-IR) and overall cancer history was observed in the general adult population, after adjusting for age, sex, and BMI. The interaction term analysis (*P* = 0.359) did not provide statistical support, indicating that, in this sample, NLR levels did not significantly amplify the carcinogenic risk cross‑sectional association of HOMA-IR. Furthermore, HOMA-IR itself, as a continuous variable, showed no significant independent cross‑sectional association with overall cancer history.

### Comparison with existing literature and interpretation

#### Implications for cardiometabolic risk stratification

From the perspective of diabetes and cardiovascular disease management, our findings carry nuanced implications. The lack of a significant synergistic effect between HOMA-IR and NLR on overall cancer history in the general population suggests that, for broad screening purposes, adding a routine inflammatory marker like NLR to an IR assessment may not substantially refine pan-cancer history prediction. This reinforces the current clinical paradigm wherein the primary focus for preventing a wide array of complications (including cancer) in patients with or at risk for T2DM/CVD remains on aggressively addressing the root causes: improving insulin sensitivity through weight management, physical activity, and glycemic control [6]. These interventions concurrently reduce systemic inflammation, thereby tackling both cardiometabolic and oncological risk pathways at their source [1].

#### Impact of cancer heterogeneity

“Overall cancer” is a highly heterogeneous endpoint. Insulin resistance and chronic inflammation have been strongly linked to specific cancer types (e.g., colorectal, liver, pancreatic, endometrial cancers). Pooling all cancers with vastly different pathological mechanisms may dilute and mask significant interaction effects that exist specifically for cancers strongly related to the metabolic-inflammatory pathway. Future research should conduct cancer type-stratified analyses to uncover potentially concealed specific cross‑sectional associations, particularly for cancers strongly linked to the metabolic-inflammatory pathway such as colorectal and liver cancers [7].

#### Specificity of inflammatory biomarkers

NLR is a practical and stable hematological indicator reflecting innate immune response and systemic inflammatory load [8]. However, the core features of chronic low-grade inflammation associated with insulin resistance often involve more specific pathways, such as adipose tissue macrophage polarization and dysregulation of adipokines and cytokines like adiponectin, leptin, interleukin-6 (IL-6), and tumor necrosis factor-alpha (TNF-α) [2]. NLR may not fully capture these more metabolism-specific inflammatory processes. A multi-dimensional assessment incorporating more specific circulating inflammatory markers, such as high-sensitivity C-reactive protein (hs-CRP) and IL-6, might better elucidate potential interaction effects.

#### Threshold and non-linear effects

The synergistic effect of IR and inflammation may not follow a simple linear relationship but could emerge only above certain thresholds of metabolic dysfunction or inflammatory burden [9, 10]. In our general-population sample, most individuals may not reach these critical thresholds, which could explain the null interaction finding. This interpretation is consistent with external evidence showing that the independent effect of NLR on cancer risk is modest (e.g., HR ~ 1.03) [10]. Collectively, these observations reinforce the notion that the epidemiologically discernible interplay between metabolism and inflammation likely manifests only in populations where both parameters reach a more pronounced pathological state. Future studies employing methods such as restricted cubic splines could formally test these potential non-linear relationships.

#### Clinical and public health implications

Our findings suggest that combining HOMA-IR and NLR for pan-cancer screening in the general population may have limited additive value. This reinforces the public health message that lifestyle interventions targeting obesity—which simultaneously improve insulin sensitivity and reduce inflammation—remain the most effective strategy for preventing both cardiometabolic diseases and associated cancers [6]. It is also important to distinguish this null finding from NLR’s established prognostic value in patients with existing cancer, highlighting that the clinical utility of inflammatory markers is highly context-dependent.

### Strengths and limitations

Cross-sectional design and the issue of reverse causation.

A key limitation of this study is its cross-sectional design. HOMA-IR and NLR were measured at the time of the NHANES examination, whereas cancer history was based on self-reported prior physician diagnosis. For participants with a history of cancer, the measured HOMA-IR and NLR may reflect metabolic and inflammatory status after cancer diagnosis or treatment, rather than before cancer onset. Therefore, the observed associations should be interpreted as cross-sectional associations only, and cannot be used to infer causality or temporal sequence. This is a critical limitation that must be considered when interpreting the null findings.

This study has several important strengths. First, the data are from the rigorously designed NHANES, providing a nationally representative sample, which allows for generalization of results to the non-institutionalized U.S. adult population. Second, key exposure variables (HOMA-IR, NLR) were based on standardized laboratory measurements, ensuring objectivity and reliability. Third, all analyses strictly followed guidelines and incorporated complex sampling weights [8], guaranteeing the accuracy of statistical inferences.

Concurrently, several additional limitations must be considered when interpreting the results. First, it should be acknowledged that our study, like many others using the NHANES database, lacks an external validation cohort. Second, the cancer outcome relied on self-report, which is susceptible to recall bias and underreporting, especially for early-stage or asymptomatic cancers. Third, although key covariates were adjusted for, the possibility of residual confounding (e.g., by factors not fully measured, such as dietary quality, physical activity levels, or specific medication history) remains. Fourth, as mentioned, the lack of data on specific cancer types and a broader panel of inflammatory markers limits a deeper exploration of mechanistic cross-sectional associations.

### Conclusion and future directions

In conclusion, this nationally representative study found no evidence that systemic inflammation, as measured by NLR, modifies the cross‑sectional association between IR and overall cancer history. The findings underscore the need for future prospective studies with cancer type‑stratified analyses and more comprehensive inflammatory marker panels to further elucidate this complex relationship.

## Supplementary Information

Below is the link to the electronic supplementary material.

Supplementary Material 1

## Data Availability

The dataset(s) supporting the conclusions of this article is(are) available in the NHANES repository, https://wwwn.cdc.gov/nchs/nhanes/.

## References

[1] Hotamisligil GS. Inflammation andmetabolic disorders. Nature. 2006;444:860–7. 10.1038/nature05485 17167474

[2] Gallagher EJ, LeRoith D. Obesity and diabetes: the increased risk of cancer and cancer-related mortality. Physiol Rev. 2015;95:727–48. 10.1152/physrev.00030.2014 PMC4491542 26084689

[3] Pollak M. The insulin and insulin-like growth factor receptor family in neoplasia: an update. Nat Rev Cancer. 2012;12:159–69. 10.1038/nrc3215 22337149

[4] Azab B, Zaher M, Weiserbs KF, et al. Usefulness of neutrophil to lymphocyte ratio in predicting short- and long-term mortality after non-ST-elevation myocardial infarction. Am J Cardiol. 2010;106:470–6. 10.1016/j.amjcard.2010.03.062 20691303

[5] Templeton AJ, McNamara MG, Šeruga B, et al. Prognostic role of neutrophil-to-lymphocyte ratio in solid tumors: a systematic review and meta-analysis. J Natl CancerInst. 2014;106:dju124. 10.1093/jnci/dju124 24875653

[6] Knowler WC, Barrett-Connor E, Fowler SE, et al. Reduction in the incidence of type 2 diabetes with lifestyle intervention or metformin. N Engl J Med. 2002;346:393–403. 10.1056/NEJMoa012512 PMC1370926 11832527

[7] Giovannucci E, Harlan DM, Archer MC, et al. Diabetes and cancer: a consensus report. Diabetes Care. 2010;33:1674–85. 10.2337/dc10-0666 PMC2890380 20587728

[8] Chen TC, Clark J, Riddles MK et al. National health and nutrition examination survey, 2015–2018: sample design and estimation procedures. Vital Health Stat 2. 2020;(184):1–31. 33663649

[9] Lou M, Luo P, Tang R, et al. Relationship between neutrophil-lymphocyte ratio and insulin resistance in newly diagnosed type 2 diabetes mellitus patients. BMC Endocr Disord. 2015;15:9. 10.1186/s12902-015-0002-9 PMC4357061 25887236

[10] Fest J, Ruiter TR, Groot Koerkamp B, et al. The neutrophil-to-lymphocyte ratio is associated with mortality in the general population: the Rotterdam Study. Eur J Epidemiol. 2019;34:463–70. 10.1007/s10654-018-0472-y PMC6456469 30569368

