# Supplementary material for: The interplay of insulin resistance and systemic inflammation on cancer history: a nationally representative cross-sectional study from NHANES
Source: Cardiovasc Diabetol Endocrinol Rep. 2026 Nov 4;12:66. doi: 10.1186/s40842-026-00335-y (PMC13732993; doi:10.1186/s40842-026-00335-y)

Supplementary Figure S1. Dose-response relationships of HOMA-IR and NLR with cancer risk using restricted cubic splines. (A) Association between HOMA-IR and the predicted log odds of cancer. (B) Association between NLR and the predicted log odds of cancer. The solid lines represent the predicted log odds, and the shaded areas indicate the 95% confidence intervals. Both associations demonstrated significant non-linearity (P for non-linearity < 0.001 for both HOMA-IR and NLR). All models are adjusted for age, sex, and BMI and incorporate NHANES complex survey weights.

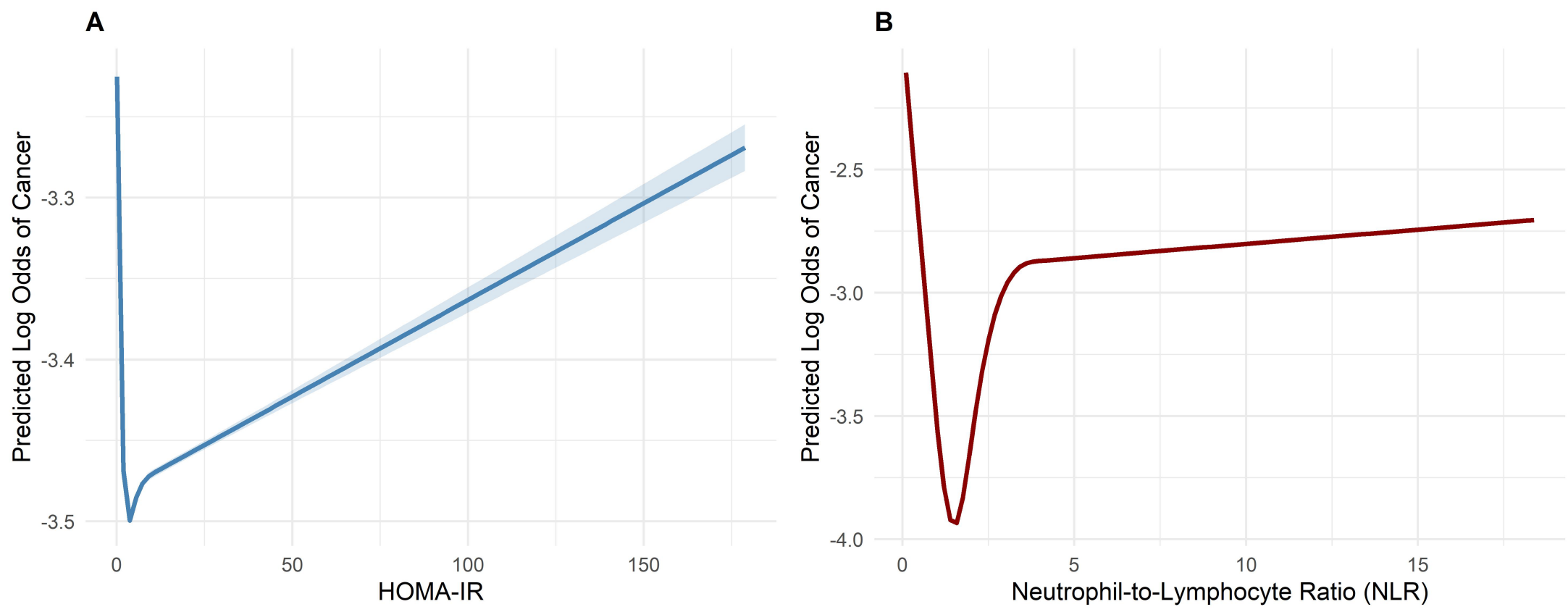

Supplement: Supplementary file 1 [file 40842_2026_335_MOESM1_ESM.pdf]
